# Reproducible changes in the anorexia nervosa gut microbiota following inpatient therapy remain distinct from non-eating disorder controls

**DOI:** 10.1080/19490976.2022.2143217

**Published:** 2022-11-18

**Authors:** Farnaz Fouladi, Emily C. Bulik-Sullivan, Elaine M. Glenny, Laura M. Thornton, Kylie K. Reed, Stephanie Thomas, Susan Kleiman, Ashlie Watters, Judy Oakes, Eun-Young Huh, Quyen Tang, Jintong Liu, Zorka Djukic, Lauren Harper, Yesel Trillo-Ordoñez, Shan Sun, Ivory Blakely, Philip S. Mehler, Anthony A. Fodor, Lisa M. Tarantino, Cynthia M. Bulik, Ian M. Carroll

**Affiliations:** a Department of Bioinformatics and Genomics, University of North Carolina at Charlotte, Charlotte, NC 28223, USA; b Department of Nutrition, Gillings School of Global Public Health, University of North Carolina at Chapel Hill, Chapel Hill, NC 27599, USA; c Department of Psychiatry, School of Medicine, University of North Carolina at Chapel Hill, Chapel Hill, NC 27599, USA; d ACUTE Center for Eating Disorders and Severe Malnutrition at Denver Health, University of Colorado School of Medicine, Denver, CO 80204, USA; e ACUTE Center for Eating Disorders and Severe Malnutrition at Denver Health, Department of Medicine, Medical Intensive Care Unit, Denver Health Hospital Authority, Denver, CO 80204, USA; f Center for Gastrointestinal Biology and Disease, School of Medicine, University of North Carolina at Chapel Hill, Chapel Hill, NC 27599, USA; g Graduate School of Professional Psychology, Morrison Family College of Health, University of St. Thomas, Minneapolis, MN, USA; h Department of Genetics, School of Medicine, University of North Carolina at Chapel Hill, Chapel Hill, NC 27599, USA; i Division of Pharmacotherapy and Experimental Therapeutics, Eshelman School of Pharmacy, University of North Carolina at Chapel Hill, Chapel Hill, NC 27599, USA; j Department of Medical Epidemiology and Biostatistics, Karolinska Institutet, Stockholm, Sweden

**Keywords:** Intestinal microbiota, anorexia nervosa, renourishment, nutrition

## Abstract

The composition of the gut microbiota in patients with anorexia nervosa (AN), and the ability of this microbial community to influence the host, remains uncertain. To achieve a broader understanding of the role of the intestinal microbiota in patients with AN, we collected fecal samples before and following clinical treatment at two geographically distinct eating disorder units (Center of Excellence for Eating Disorders [UNC-CH] and ACUTE Center for Eating Disorders [Denver Health]). Gut microbiotas were characterized in patients with AN, before and after inpatient treatment, and in non-eating disorder (non-ED) controls using shotgun metagenomic sequencing. The impact of inpatient treatment on the AN gut microbiota was remarkably consistent between eating disorder units. Although weight in patients with AN showed improvements, AN microbiotas post-treatment remained distinct from non-ED controls. Additionally, AN gut microbiotas prior to treatment exhibited more fermentation pathways and a lower ability to degrade carbohydrates than non-ED controls. As the intestinal microbiota can influence nutrient metabolism, our data highlight the complex microbial communities in patients with AN as an element needing further attention post inpatient treatment. Additionally, this study defines the effects of renourishment on the AN gut microbiota and serves as a platform to develop precision nutrition approaches to potentially mitigate impediments to recovery.

## Introduction

The intestinal microbiota is a malleable complex community of microorganisms residing in the mammalian gastrointestinal (GI) tract, which is profoundly shaped by multiple host and environmental factors including genetics, age, sex, geographical location, xenobiotics, and nutrition.^1^ Preclinical studies and human clinical trials support the concept that this complex microbial community plays a significant role in calorie harvest from the diet and subsequent host metabolism.^2^ Specifically, mice raised in the absence of microbial associates (germ-free [GF] mice) consume approximately 30% more daily kilocalories (kcal) than mice harboring an intestinal microbiota, yet accumulate less body fat.^3^ Additionally, targeting the gut microbiota with antibiotics promotes stool calorie loss (a proxy for decreased energy harvest from the intestinal lumen) in humans.^2^ These studies highlight the potential for gut microbial communities to influence host dietary metabolism.

As diet significantly affects the composition of the intestinal microbiota,^4^ and calorie restriction impacts energy extraction from the gut,^2^ the influence that complex microbial communities have on eating disorders—specifically patients with anorexia nervosa (AN)—is an intriguing area of research. To date, multiple studies have identified distinct patterns in the gut microbiota between patients with AN and healthy individuals.^5–12^ Additionally, changes in the intestinal microbiota before and after clinical renourishment in patients with AN have been reported, with the composition of the gut microbiota remaining distinct from non-eating disorder (non-ED) controls.^5,7,10,13^ Several studies have also identified metabolites produced by gut microbial communities that differ between patients with AN and controls,^14–16^ suggesting changes in the intestinal microbiota may have a functional influence on the host. These studies have relied on 16S rRNA gene sequencing to profile the gut microbiota—overlooking functional genomic metabolic pathway data. Our current study advances this field by characterizing gut microbial communities in patients with AN prior to and following clinical renourishment at two geographically distinct eating disorder treatment clinics using shotgun metagenomic sequencing.

## Results

### Impact of inpatient treatment on the gut microbiota from patients with AN

**Figure 1.**
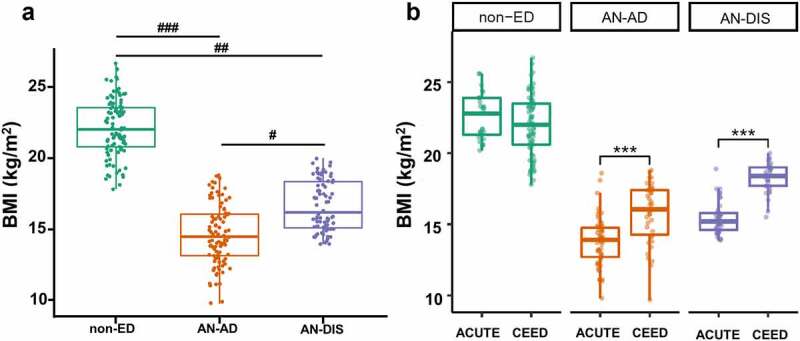
Patients with AN gain significant weight following inpatient treatment. Boxplots showing BMIs (a & b) of non-ED controls and patients with anorexia nervosa (AN) before (AD) and after (DIS) inpatient treatment combined (a) and separated by clinical site (b). Statistical tests: unpaired *t*-test for non-ED versus AN-AD and non-ED versus AN-DIS, paired *t-*test for AN-AD versus AN-DIS. ^###^ *p* = 2.4 × 10^−61^, ^##^*p* = 2.53 × 10^−44^, ^#^*p* = 1.02 × 10^−15^, *** *p* < .0001.

Patients with AN were admitted to the inpatient unit at the UNC Center of Excellence for Eating Disorders (CEED) (*N* = 38) or Denver ACUTE (*N* = 55) with treatment spanning 4–10 and 1–7 weeks, respectively. Body mass indices (BMIs) of non-ED controls (*N* = 98: CEED *N* = 69, ACUTE *N* = 29) were significantly higher than patients with AN both prior to and following clinical renourishment (Figure 1 a and b). Average baseline BMI for patients with AN was 14.6 ± 2.12 kg/m^2^ (CEED: 15.74 ± 2.11 kg/m^2^, ACUTE: 13.78 ± 1.74 kg/m^2^, Figure 1 and Table 1). Of note, ACUTE specializes in medical stabilization of individuals with more severe AN presentations,^17^ reflected in their significantly lower BMIs at admission (Figure 1b). At each program, kilocalories were incrementally increased, resulting in an average intake of 3,200 (CEED) and 3,400 (ACUTE) kcal/d by discharge. Patients gained significant weight with an average increase of 5.33 ± 4.43 kg (CEED: 6.27 ± 5.13 kg; ACUTE: 4.63 ± 3.75 kg) (Table 1). Upon discharge, 60 patients (CEED = 17, ACUTE = 43) had a BMI (13.9–18.4 kg/m^2^) in the underweight range (i.e., <18.5 kg/m^2^), with the remaining 16 (CEED = 15, ACUTE = 1) patients attaining a BMI above this threshold (18.5–20 kg/m^2^). As a medical stabilization program for patients with extreme AN, ACUTE does not retain patients until weight is fully restored, but only until they are able to admit to residential eating disorder treatment centers. Despite gaining substantial weight, the mean weight for patients at discharge was still significantly lower than the mean weight of non-ED controls (44.9 ± 5.63 kg vs 60.7 ± 7.29 kg, Table 1).

**Table 1.** Characteristics of non-ED controls and patients with anorexia nervosa at CEED and ACUTE eating disorder units.

	Non-ED		AN-AD		AN-DIS	
	CEED	ACUTE	CEED	ACUTE	CEED	ACUTE
Number	69	29	38	55	33	45
BMI (kg/m^2^)	21.98 ± 2.13	22.56 ± 1.60	15.74 ± 2.11	13.78 ± 1.74	18.28 ± 1.05	15.41 ± 1.10
Age	23.6 ± 5.28	31.1 ± 5.64	23.2 ± 6.87	27.7 ± 7.41	22.6 ± 6.90	27.4 ± 6.68
Weight (kg)	59.73 ± 7.18	62.85 ± 7.21	42.56 ± 7.26	37.30 ± 5.63	49.34 ± 4.56	41.73 ± 3.92
Recovery time (d)	–	–	–	–	32.37 ± 26.21	17.66 ± 9.56
BMI change (kg/m^2^)	–	–	–	–	2.36 ± 1.97	1.71 ± 1.42
BMI change per day (kg/m^2^.d)	–	–	–	–	0.07 ± 0.03	0.09 ± 0.05
Weight change (kg)	–	–	–	–	6.27 ± 5.13	4.63 ± 3.75
Weight change per day (kg/d)	–	–	–	–	0.20 ± 0.08	0.24 ± 0.15
Missing weight data	–	–	–	1	1	1

**Figure 2.**
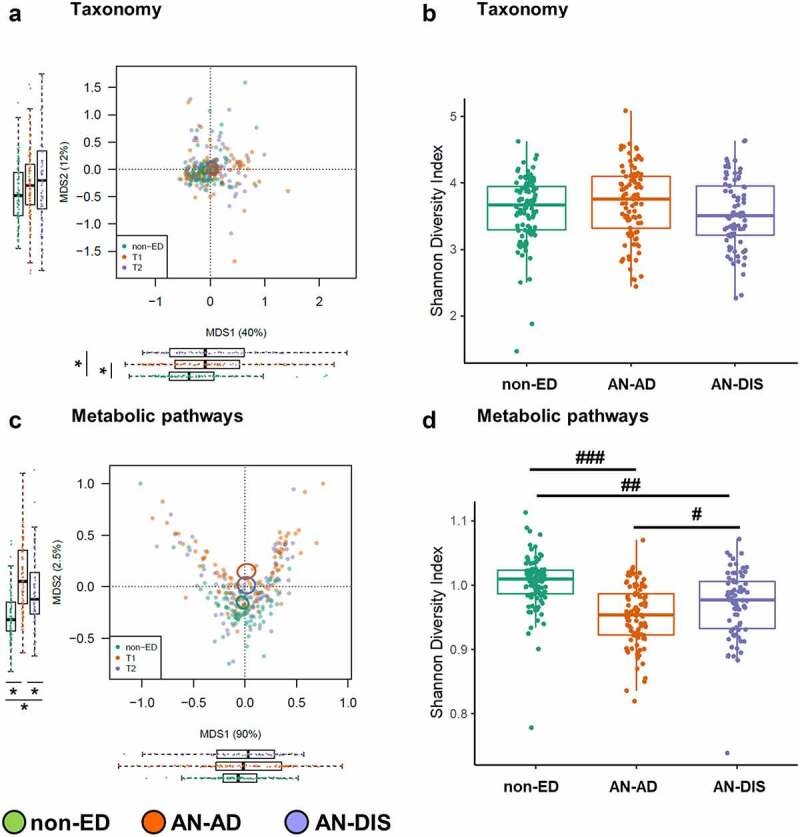
Patients with AN exhibit distinct gut microbiomes, before and following inpatient treatment, compared to non-ED controls. Principal Coordinates Analysis plot using Bray–Curtis distances (a) and Shannon diversity (b) based on species-level taxonomies. Principal Coordinates Analysis plot using Bray–Curtis distances (c) and Shannon diversity (d) based on metabolic pathways. Boxplots adjacent to each plot show the first and second axes (MDS1 and MDS2) for non-ED and AN (AD and DIS) gut microbiomes. Tests: unpaired *t*-test for non-ED versus AN (AD and DIS), paired *t*-test for AD versus DIS. ^###^*p* = 5.2 × 10^−13^, ^##^*p* = 9.39 × 10^−6^, ^#^*p* = .004, *p < .05.

Gut microbiotas from patients with AN and non-ED controls clustered separately across the first axis of the ordination plot (MDS1) based on taxonomic composition generated via shotgun metagenomic data (Figure 2a, *t*-test across MDS1 non-ED vs AN adjusted *p* < .01). However, gut microbiotas obtained from patients with AN upon admission and discharge were not distinct (*t*-test across MDS1 AN-AD vs AN-DIS adjusted *p* = .50). Taxonomic separation between AN and control groups across MDS1 was driven by samples obtained from the Denver ACUTE center, as distinct clusters were only found in samples obtained from this site when sequencing data from each clinical site were analyzed independently (supplemental Figure 1A and E). The gut microbiotas from patients with AN prior to treatment and non-ED controls were also distinct based on predicted functional metabolic pathways, with samples from AN-AD and AN-DIS clustering separately (Figure 2c, *t*-test across MDS2 non-ED vs AN-AD, non-ED vs AN-DIS, and AN-AD vs AN-DIS adjusted *p* < .01). In contrast to the taxonomy-based analysis, distinct clusters between AN patients and non-ED controls were consistent at both clinical sites across the MDS2 axis (supplemental Figure 1C and G).

Shannon diversity based on taxonomy was similar between patients with AN (AN-AD and AN-DIS) and non-ED controls, irrespective of clinical site (Figure 2b, supplemental Figure 1B and F). In contrast, Shannon diversity based on metabolic pathways was significantly lower in AN-AD than non-ED controls at both sites (Figure 2d, supplemental Figure 1D and H; adjusted *p* < .001). Inpatient treatment was associated with increased diversity of microbial biochemical pathways yet remained lower than non-ED controls (Figure 2d, AN-AD vs AN-DIS adjusted *p* = .004, AN-DIS vs non-ED adjusted *p* < .001), further suggesting that the gut microbiota of patients with AN following inpatient treatment does not represent the gut microbiota of non-ED individuals.

### Inpatient treatment alters gut microbial metabolism

As we found that gut microbiotas between AN and non-ED groups separated based on taxonomy, we next investigated which specific bacterial groups were driving this separation. Consistent with our initial observations, approximately 1,500 taxa differed in relative abundance between patients with AN (AN-AD and AN-DIS) and controls, while only 25 taxa differed between AN-AD and AN-DIS (supplemental table 1). Of these taxa, *Bifidobacterium adolescentis, Faecalibacterium prausnitzii, Flavonifractor plautii, Collinsella aerofaciens, Ruminococcus bicirculans*, and unclassified *Ruminococcaceae* and *Lachnospiraceae* families were the most abundant groups elevated in non-ED controls. As we found that samples obtained from Denver ACUTE drove the taxonomic separation in gut microbiotas between AN and control groups (supplemental Figure 1A and E), we investigated whether taxonomic differences existed in clinical groups (i.e., AN-AD, AN-DIS, and non-ED controls) between the UNC CEED and Denver ACUTE sites. Interestingly, *Bifidobacterium animalis* was the only species found to differ between clinical sites with AN-DIS samples, and no differences were found between clinical sites with AN-AD samples. These data further suggest that the composition of the intestinal microbiotas differs between patients with AN and non-ED controls, and that clinical treatment, as operationalized at these centers, does not fully restore a healthy gut microbiota in these patients (i.e., a gut microbiota similar to a non-ED control).

**Figure 3.**
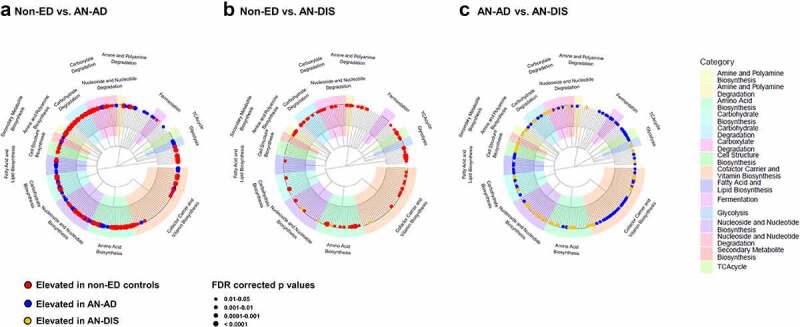
Inpatient treatment changes the metabolic potential of gut microbiomes from patients with AN. Gut microbiome functional capacity is distinct between non-ED controls and patients with AN prior to renourishment (a); non-ED controls and patients with AN post inpatient treatment (b); and patients with AN prior to and post inpatient treatment (c). Trees display the hierarchy of metabolic pathways.

Forty-four functional metabolic pathways within the intestinal microbiota were higher in abundance in patients with AN at admission (AN-AD) compared with non-ED controls—with 86 pathways higher in abundance in non-ED controls compared with AN-AD (Figure 3a, supplemental Table 2). The intestinal microbiotas from AN-AD samples exhibited a notable increase in the relative abundance of fermentation pathways. Following inpatient treatment, the number of elevated metabolic pathways in patients with AN compared to non-ED controls decreased to 8 (Figure 3b). Moreover, 119 metabolic pathways exhibited differential abundances between patients with AN prior to and post renourishment (68 elevated in AN-AD and 51 elevated in AN-DIS, Figure 3c). Together these data suggest a restoration of some metabolic processes in the gut microbiota of patients with AN following clinical treatment. However, when investigating the two clinical sites (UNC CEED and Denver ACUTE) independently, the differences in the metabolic pathways between AN-AD and AN-DIS did not survive multiple hypothesis testing, possibly due to decreased sample size (supplemental Figure 2A-F).

### Inpatient treatment causes consistent changes to gut microbial communities in patients with AN

**Figure 4.**
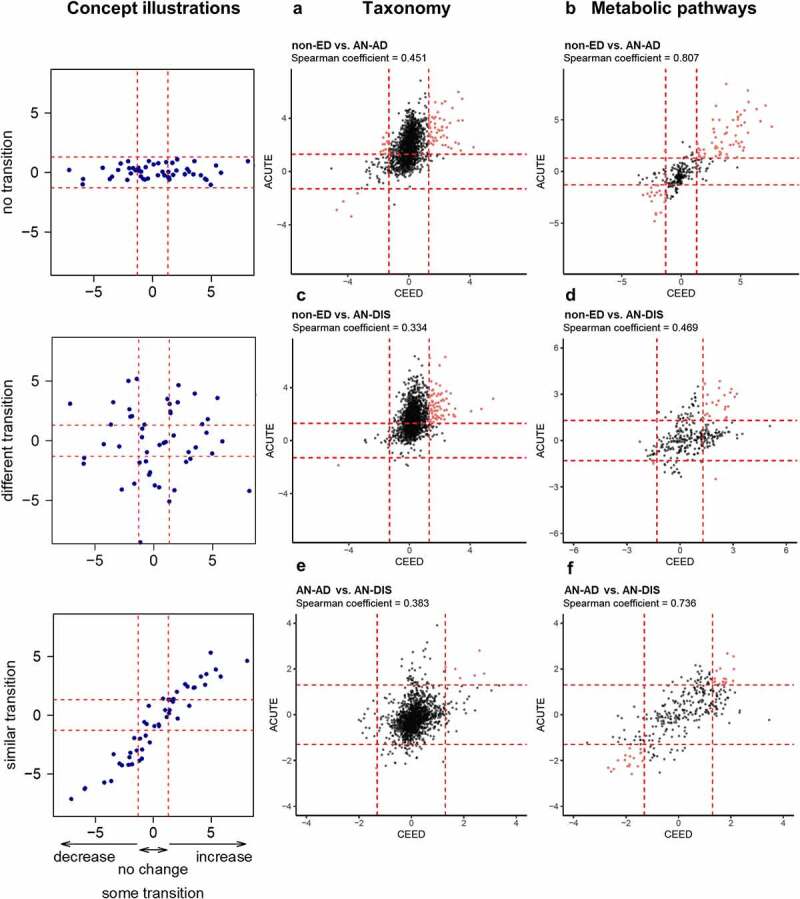
Changes associated with inpatient treatment are consistent between geographically distinct eating disorder units. Scatter plots showing the unadjusted log_10_ *p*-values from *t*-tests comparing the log_10_ normalized counts of genera (a, c, e) and relative abundances of metabolic pathways (b, d, f) in non-ED controls versus patients with anorexia nervosa prior to inpatient treatment (non-ED vs AN-AD), non-ED controls versus patients with anorexia nervosa following inpatient treatment (non-ED vs AN-DIS), and patients with anorexia nervosa prior to versus following inpatient treatment (AN-AD vs AN-DIS) at CEED and ACUTE. Genera and metabolic pathways with unadjusted *p*-values of less than 0.05 at both CEED and ACUTE are colored red. Cartoon concept depicts situations of perfect and random associations.

As we observed more robust differences in gut microbial communities between controls and patients with AN at Denver ACUTE compared with UNC CEED, we hypothesized that the taxonomic and metabolic changes in the gut microbiota are unique to each site. We investigated this hypothesis by correlating log_10_ *p* values for taxonomy and metabolic pathways as previously described.^18^ In contrast to this concept, we observed significant correlations in gut microbiota changes between CEED and ACUTE eating disorder units. Log_10_ *p* values for changes in genus abundance between (I) AN-AD and non-ED controls (Spearman coefficient = 0.45, adjusted *p* = 3.01 × 10^−84^), (II) AN-DIS and non-ED controls (Spearman coefficient = 0.33, adjusted *p* = 9.3 × 10^−45^), and (III) AN-AD and AN-DIS (Spearman coefficient = 0.38, adjusted *p* = 2.16 × 10^−59^, Figure 4 a, 4 and e). Additionally, more robust correlations were observed when comparing the same groups using functional metabolic pathways (Figure 4 b, 4 and f). These robust correlations suggest that although gut microbiota changes in patients with AN treated at ACUTE are more notable, the taxonomic and metabolic pathways that change during the course of treatment in patients with AN—and the differences from non-ED controls—have a consistent trend at both sites. The greater the severity of the disease and the larger sample size of patients at ACUTE compared with CEED could explain why more significant differences between the gut microbial communities of non-ED and controls were driven by patients from the ACUTE eating disorder unit.

## Discussion

Our study reports the use of shotgun metagenomic sequencing to characterize the intestinal microbiota in patients with AN prior to and following inpatient treatment at two geographically distinct eating disorder specialist units. We report that the gut microbiota harbored in patients with AN is distinct from non-ED controls—a finding consistent with previous 16S rRNA gene-based analyses.^5–12^ Interestingly, the distinction between groups of gut microbiotas was clearer when using metabolic pathway data than taxonomic data—suggesting that “what genes are present” rather than “what microbes are present” provides a clearer picture of predicted functional changes in the gut microbiota following inpatient treatment of patients with AN.

One of the notable observations from our study was the consistency of gut microbiota changes following treatment across clinical sites. Specifically, the taxonomic and metabolic changes in the gut microbiota in patients with AN prior to and following treatment were strikingly similar. This finding is of particular interest as each eating disorder unit has differing approaches to renourishment and treatment of patients with AN. Specifically, CEED’s approach is to initiate renourishment at 1100–1500 kcal/d (starting calories consider intake weight, pre-hospitalization caloric intake, resting energy expenditure, and clinical features) and aims for 50% carbohydrates, 20–25% protein, and 30% fat based on the Diabetic Exchange System. Target weight for adults is set at 80–85% of ideal body weight (IBW) based on Metropolitan Life Tables.^19^ Calories are increased by 300 kcal every 1–3 d while aiming for an increase of 1–2 kg/week. In contrast, ACUTE’s approach to renourishment is to initiate refeeding at 1600 kcal/d with a diet containing a macro-composition of 40% carbohydrate, 20% protein, and 40% fat. Calories are increased by 300–400 kcal every 3–4 d to achieve a desired weight gain of 1.6–1.8 kg/week with a target weight set at 70% of IBW. Consistent gut microbial changes at both clinical sites suggest that patients with AN possess a gut microbiota similar in composition and metabolic potential that responds to inpatient treatment in a predictable pattern. Although the gut microbiota’s response to treatment is consistent, it remains distinct to non-ED controls suggesting that initial inpatient clinical renourishment does not return the gut microbiota to a healthy state. As relapse rates for AN are high,^20^ and as the intestinal microbiota is closely tied to adiposity,^2,3^ it is tempting to speculate that continued attention to gut microbes may play a role in maintaining a healthy weight post discharge from an eating disorders specialist unit.

Based on data generated by this study, it appears that the intestinal microbiota of patients with AN are metabolically dysfunctional—a phenomenon that is not rectified with inpatient clinical treatment. In particular, an enrichment in carbohydrate degradation, cell structure biosynthesis, and amine and polyamine biosynthesis was observed in non-ED controls compared to patients with AN at hospital admission. Additionally, the gut microbiome of patients with AN prior to renourishment exhibits an enrichment in fermentation-related pathways. This finding suggests a preference for the AN gut microbiome to metabolize indigestible carbohydrates (fiber) as an energy source—possibly reflecting the preference of patients with AN to consume a high-fiber, low-sugar diet. Moreover, dysregulation of cell structure, amine, and polyamine biosynthesis pathways suggests a gut microbiota in patients with AN that is defective in key functions for maintaining a healthy microbial ecosystem—possibly due to a lack of nutrients. Interestingly, a recent study investigating metabolic pathways of the gut microbiome of individuals consuming a severe calorie-restricted diet (approximately 60% restriction) reported an increase in carbohydrate metabolism, suggesting the metabolic status of the AN gut microbiome is not merely a result of caloric restriction.^21^ Following inpatient treatment, the gut microbiome of patients with AN exhibits fewer dysregulated metabolic pathways; however, non-ED controls were still enriched for carbohydrate degradation, and amine and polyamine biosynthesis, suggesting a partial restoration in the metabolic pathways of the AN gut microbiome.

Although our study does not address causality, recent studies transplanting fecal samples from patients with AN and non-ED controls into GF mice have addressed this phenomenon with mixed results.^11,22^ The data reported here complement these studies by identifying specific bacterial taxa that may have a causative influence on host adiposity. Specifically, *Bifidobacterium adolescentis* and *Faecalibacterium prausnitzii*, depleted in patients with AN, are potential candidates to be used in gnotobiotic studies (mono-/dual-association or a consortium of microbes) coupled with calorie restriction, refeeding, or precision nutrition experiments to examine the contribution of enteric microbes to intestinal dysfunction in the context of nutrient deprivation. As our study identified certain taxa and metabolic pathways that distinguish the intestinal microbiota in patients with AN from non-ED controls, our findings provide a foundation that will potentially have an impact on treatment of this illness. Specifically, it is possible that the taxa and metabolic pathways we identify play a mechanistic role in weight gain during the clinical renourishment of patients with AN. If these microbes prove to be beneficial or detrimental to renourishment, then targeted approaches to increase or decrease their abundance prior to or during clinical renourishment may lead to safer and more efficient interventions for AN. One potential approach to accomplish this is via precision nutrition during renourishment of patients with AN.^23^ The premise of this approach emerges from the use of microbiota-directed complementary foods (MDCF) in children presenting with moderate acute malnutrition (MAM). Historically, weight gain in MAM has been approached by increasing caloric consumption without careful consideration of the nutritional composition of the food used for renourishment. MDCFs aim to restore the abundance of enteric microbes reported to be diminished in children with MAM^24^ and have yielded significantly greater weight gain and better weight-for-length and weight-for-age *z*-scores, while consuming fewer calories, than Bangladeshi children nourished with traditional ready-to-use-supplementary foods.^25^ Since nutrient deprivation is central to both MAM and AN, a precision nutrition approach using MDCFs to renourish patients with AN is a worthwhile approach to explore.^26^ An MDCF for the treatment of AN could potentially restore a normal microbial ecosystem, promote a more functional intestine, and ultimately improve both the efficacy and tolerability of renourishment in AN by maximizing efficiency and decreasing the sheer quantity of food necessary to achieve weight restoration. Longer-term MDCF interventions could adapt to a normalizing microbial ecosystem, supporting the maintenance of regained weight and reduced risk of relapse.

## Methods

### Recruitment of patients

This study received approval from the Biomedical Institutional Review Board at the University of North Carolina at Chapel Hill (UNC IRB: 14–0045, Colorado Multiple Institutional Review Board (COMIRB) #16-1514, ClinicalTrials.gov: NCT03119272). Written consent was provided by all participants before study participation, with parental permission forms and age-appropriate assent forms provided to participants younger than 18 y.

Eligible inpatient participants were evaluated by trained professionals at the UNC CEED or the ACUTE Center for Eating Disorders and Severe Malnutrition at Denver Health and met DSM-5 criteria for AN.^27^ Non-ED control recruitment was carried out via university flyers, and listser vs non-ED controls had no history of either a BMI outside 18.5–24.9 kg/m^2^ or any eating disorder (Table 1). Exclusion criteria for all study participants were based on factors that influence the composition of the intestinal microbiota, which include: history of GI tract surgery (other than cholecystectomy) or any clinical diagnosis that could explain chronic or recurring bowel symptoms (e.g., inflammatory bowel diseases, irritable bowel syndrome, or celiac disease, treatment in the previous 2 months with antibiotics, nonsteroidal anti-inflammatory drugs, or steroids, and intentional use of probiotics in the previous 2 months).

Stool sample collection from patients with AN and non-ED controls has been previously described.^13^ Briefly, patients with AN provided a stool sample at admission to CEED or ACUTE (AD) and discharge from CEED or ACUTE after clinical renourishment (DIS). Non-ED controls provided a single stool sample. All samples were stored at 4°C until they were either transported (AN samples) or shipped overnight with ice packs (non-ED samples) to laboratories at UNC-CH or Denver Health. Upon arrival at the laboratory, fresh stool samples were immediately mechanically homogenized, aliquoted into 2-mL cryovials, and stored at −80°C for future microbiota characterization. Non-ED control samples were collected at home using a stool collection kit and stored at 4°C until they were shipped overnight with ice packs to our laboratory. Exposure of human stool samples to ambient air and extended periods at 4°C may influence the composition of the fecal microbiota. We attempted to limit these exposures to maintain the integrity of fecal microbial communities.

### DNA isolation and shotgun metagenomic sequencing

DNA was isolated from human fecal samples using a combination of physical disruption of bacterial cells and phenol-chloroform extraction, followed by a DNA clean-up kit (Qiagen DNeasy Blood and Tissue extraction kit, Valencia, CA), as previously described.

### Metagenomic analysis

Shotgun metagenomic reads were sequenced on the Illumina NovaSeq 6000 Platform (Illumina, San Diego, CA) at the UNC-CH high-throughput sequencing facility in the Carolina Center for Genome Sciences at the UNC School of Medicine using 2 × 150 basepair reads. Adapters were removed using Trimmomatic.^28^ In addition, reads were cut when the average quality score within a 4-base wide sliding window dropped below 20 and reads with less than 50 base long were removed. Human genomic reads were discarded using KneadData

(http://huttenhower.sph.harvard.edu/kneaddata).

Taxonomic profile was characterized using Kraken2,^29^ through an automated pipeline BioLockJ (https://github.com/BioLockJ-Dev-Team/BioLockJ), and metabolic profiles were characterized through the HUMAnN2 pipeline.^30^

### Statistics

To account for different sequence depth across samples, taxonomic tables were normalized using the following formula^31^:

$$\log_{10}\left(\left[\begin{array}{c} \frac{Raw\;count\;\epsilon \;sample\left(i\right)}{\#\;of\;sequences\;\epsilon \;sample\;\left(i\right)}\times Average\;\;\#\;\;of\;sequences\;per\;sample \end{array}\right]+1\right)$$

Taxa that had reads less than one millionth of total reads or were present in less than 25% of samples were removed. Metabolic pathways were normalized to copies per million and were removed if they were present in less than 25% of samples. Shannon diversity index, a measurement of richness and evenness, was calculated using the vegan package in R. Principal Coordinates Analysis using the Bray–Curtis distance was used to visualize between-samples differences in taxonomic and metabolic pathway compositions using the “capscale” function in the vegan package in R. Unpaired and paired *t*-tests were used to compare the normalized count of taxonomies or metabolic pathways between non-ED controls and AN patients and AN-AD and AN-DIS patients, respectively. *p*-Values were corrected for multiple hypothesis testing using the Benjamini Hochberg procedure. Spearman rank-order correlations were performed between log_10_ *p*-values obtained from *t*-tests comparing taxa or metabolic pathways in non-ED vs AN-AD, non-ED vs AN-DIS, and AN-AD vs AN-DIS. *p*-Values for taxa or metabolic pathways that were higher in non-ED controls compared to AN or were higher in AN-AD compared to AN-DIS were multiplied by −1 after log_10_ transformation. All statistics were performed in R version 4.0.2.

## Supplementary Material

Supplemental Material

## Additional information

Correspondence and requests for materials should be addressed to Ian Carroll, ian_carroll@med.unc.edu.

## Data sharing statement

Data and codes are available at https://github.com/FarnazFouladi/Anorexia_Analyses2021.
