# Supplementary figures and images for: Reproducible changes in the anorexia nervosa gut microbiota following inpatient therapy remain distinct from non-eating disorder controls
Source: Gut Microbes. 2022 Nov 18;14(1):2143217. doi: 10.1080/19490976.2022.2143217 (PMC9678007; doi:10.1080/19490976.2022.2143217)

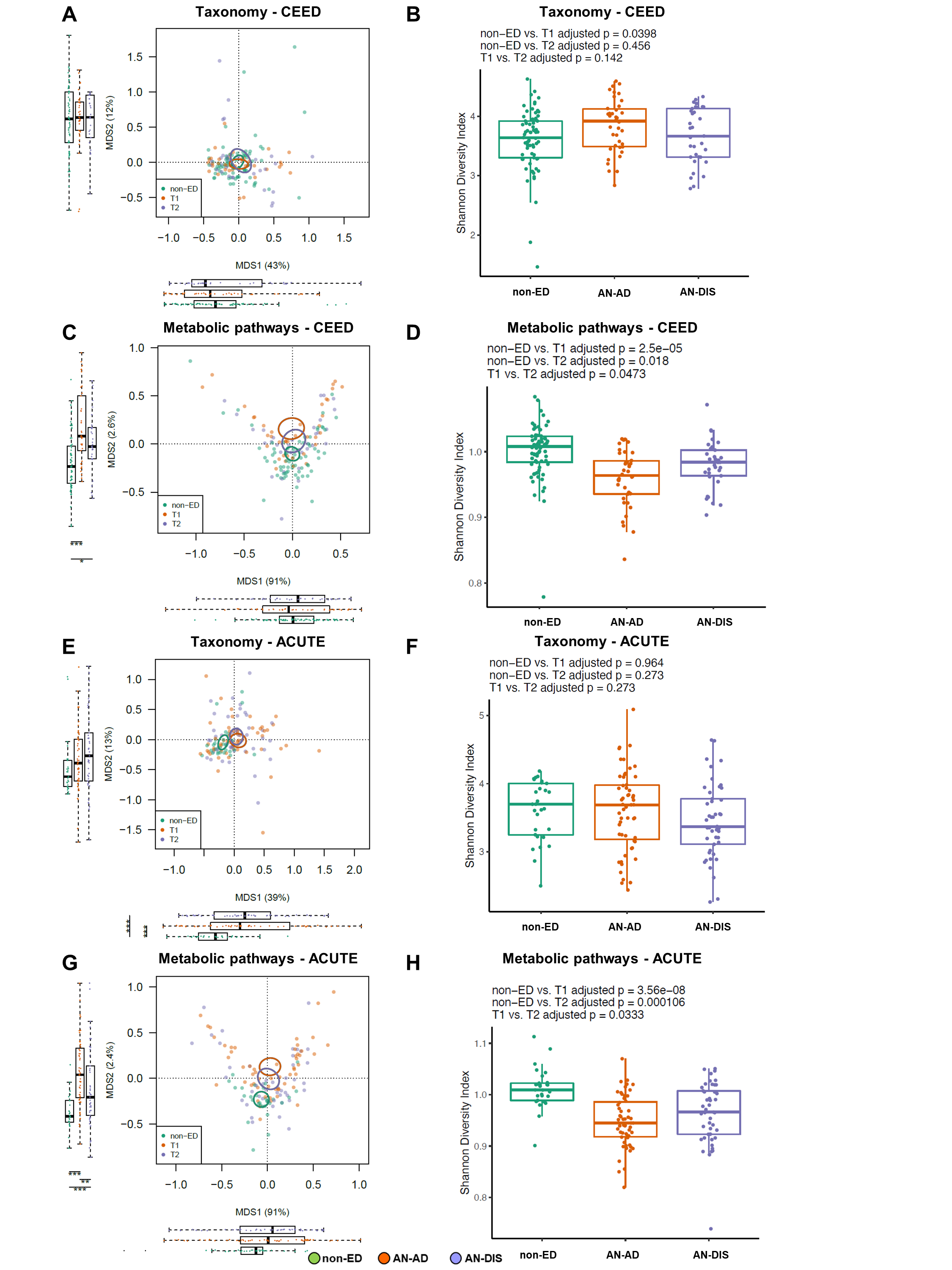

Supplement: Supplemental Material [file KGMI_A_2143217_SM3225.zip › Supp Fig 1.tif]

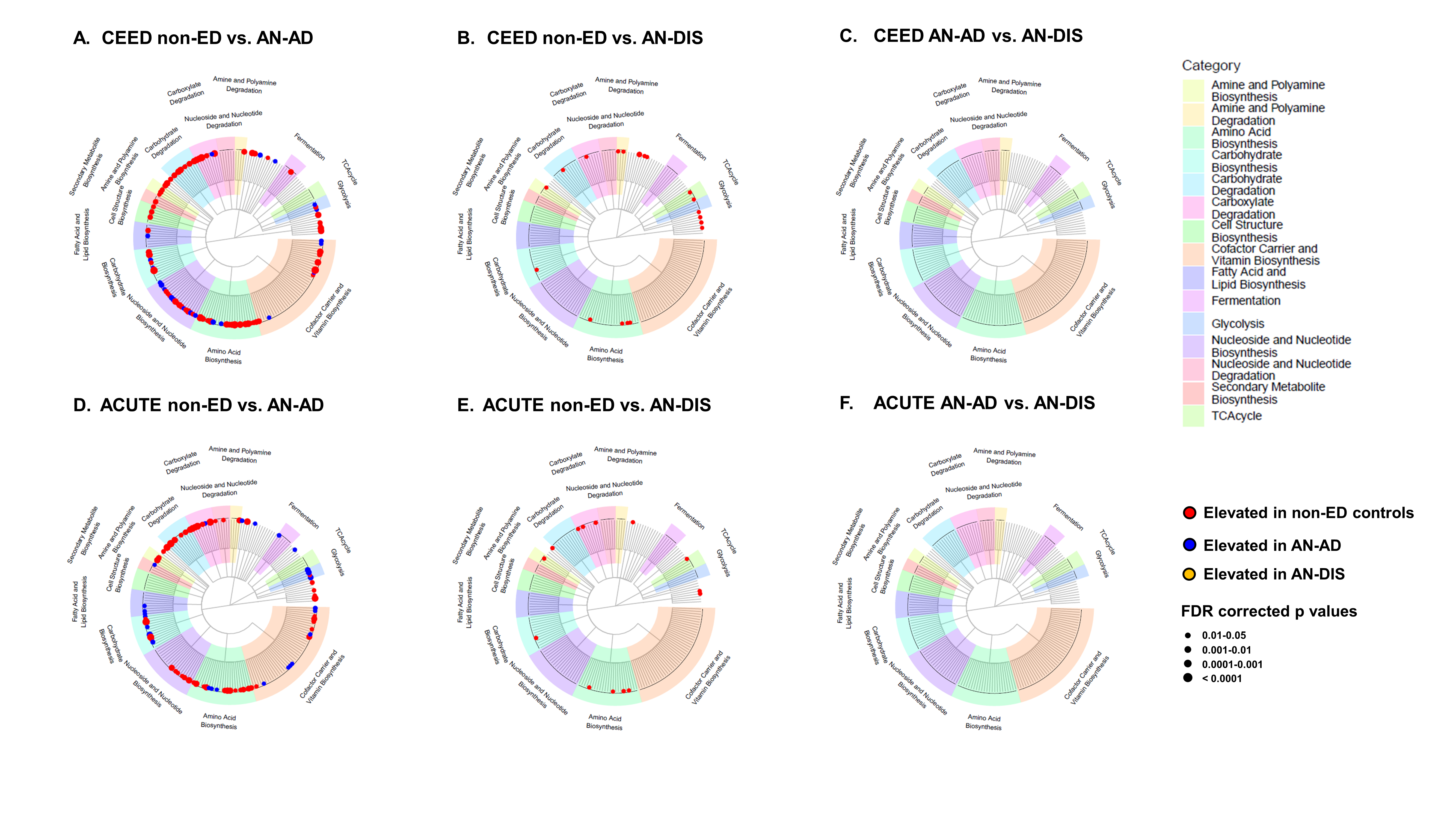

Supplement: Supplemental Material [file KGMI_A_2143217_SM3225.zip › Supp Fig 2.tif]
